# Involvement of cytokinins in STOP1-mediated resistance to proton toxicity

**DOI:** 10.1007/s44154-022-00033-6

**Published:** 2022-03-08

**Authors:** Fei Jiang, Sangbom M. Lyi, Tianhu Sun, Li Li, Tao Wang, Jiping Liu

**Affiliations:** 1 Robert W. Holley Center for Agriculture and Health, USDA-ARS, Ithaca, NY 14853 USA; 2 Chengdu Institute of Biology, Chinese Academy of Sciences, Chengdu, Sichuan China; 3 Plant Breeding and Genetics Section, School of Integrative Plant Science, Cornell University, Ithaca, NY 14853 USA

**Keywords:** Arabidopsis, Cytokinin, Cytokinin signaling output, Low pH stress, Proton stress, Resistance, STOP1, Tolerance

## Abstract

STOP1 (sensitive to proton rhizotoxicity1) is a master transcription factor that governs the expression of a set of regulatory and structural genes involved in resistance to aluminum and low pH (*i.e*., proton) stresses in Arabidopsis. However, the mechanisms and regulatory networks underlying STOP1-mediated resistance to proton stresses are largely unclear. Here, we report that low-pH stresses severely inhibited root growth of the *stop1* plants by suppressing root meristem activities. Interestingly, the *stop1* plants were less sensitive to exogenous cytokinins at normal and low pHs than the wild type. Significantly, low concentrations of cytokinins promoted root growth of the *stop1* mutant under low-pH stresses. Moreover, lateral and adventitious root formation was stimulated in *stop1* and by low-pH stresses but suppressed by cytokinins. Further studies of the expression patterns of a cytokinin signaling reporter suggest that both the loss-of-function mutation of STOP1 and low-pH stresses suppressed cytokinin signaling outputs in the root. Furthermore, the expression of critical genes involved in cytokinin biosynthesis, biodegradation, and signaling is altered in the *stop1* mutant in response to low-pH stresses. In conclusion, our results reveal a complex network of resistance to low-pH stresses, which involves coordinated actions of STOP1, cytokinins, and an additional low-pH-resistant mechanism for controlling root meristem activities and root growth upon proton stresses.

**Supplementary Information:**

The online version contains supplementary material available at 10.1007/s44154-022-00033-6.

## Highlight

We demonstrate that low-pH (proton) stresses and STOP1 functions affect cytokinin signaling outputs, which in turn regulate root growth. Cytokinins play crucial roles in STOP1-mediated resistance to proton stresses.

## Introduction

In Arabidopsis, *STOP1* (*sensitive to proton rhizotoxicity*) encodes a master transcription factor that regulates a set of genes critical for resistance to a range of abiotic stresses, including aluminum (Al) toxicity and low-pH (*i.e*., proton) stresses (Iuchi et al., 2007; Koyama et al., 2021; Sadhukhan et al., 2021). Therefore, the loss-of-function *stop1* mutants are susceptible to low-pH and Al stresses (Iuchi et al., 2007; Liu et al., 2009). Although it has been well documented that STOP1 confers Al resistance via controlling the expression of a set of Al-resistant genes, including *ALMT1*, *MATE*, and *ALS3* (Liu et al., 2009; Sawaki et al., 2009), the mechanisms underlying STOP1-mediated resistance to proton stresses remain unclear (Liu et al., 2009; Liu et al., 2012).

Cytokinins are a class of phytohormones that regulate various developmental and physiological processes, including cell division, shoot and root development, and responses to pathogens (Mok and Mok, 2001; Werner et al., 2003; Müller, 2011; Kieber and Schaller, 2018). Although cytokinins control cell proliferation in meristems, their actions in the root and shoot meristems are opposite (Werner et al., 2001; Werner et al., 2003). Cytokinins are positive regulators of cell division and meristem activities in the shoot apical meristem. Thus, they promote shoot growth and the formation of inflorescence meristems and leaf primordia (Werner et al., 2003). In contrast, cytokinins negatively regulate root growth and development as they suppress cell division in the root apical meristem and inhibit the formation of lateral root and adventitious root primordia (Werner et al., 2003; Dello Ioio et al., 2007). Lateral roots emerge from the root pericycle, while adventitious roots arise from an organ other than the root, such as a stem.

In plants, cytokinin homeostasis is maintained by de novo biosynthesis, import/export, formation of conjugates, and catabolism (Mok and Mok, 2001). In Arabidopsis, the cytokinin-mediated responsive and regulatory functions are facilitated via a two-component signaling (TCS) system where the initial cytokinin signals are perceived by the membrane-localized two-component histidine-kinase receptors, AHK2, AHK3, and AHK4 (AHK, Arabidopsis Histidine Kinase). Then, the signals are further transduced through a series of His-Asp phosphorelay events that lead to the activation of a set of transcription factors in the nucleus, which modulates the expression and function of different response regulators (RRs), leading to the final realization of the cytokinin signaling (Hwang and Sheen, 2001; Ferreira and Kieber, 2005; To and Kieber, 2008; Cheng and Kieber, 2014).

Although cytokinins have been implicated in plant resistance to abiotic stresses, including cold, osmotic, drought, and salt stresses (Tran et al., 2007; Argueso et al., 2009; Ha et al., 2012; O'Brien and Benková, 2013; Zwack and Rashotte, 2015), their roles in resistance to low-pH stresses remain unknown. Here, we report that both STOP1 functions and proton stresses suppress cytokinin signaling outputs in the root, influencing root growth under low-pH stresses. Therefore, our results reveal a complex network involving cytokinins in STOP1-mediated resistance to low-pH stresses in Arabidopsis.

## Results

### Loss-of-STOP1 function and low-pH stresses reduce root sensitivity to cytokinin inhibition

**Fig. 1:**
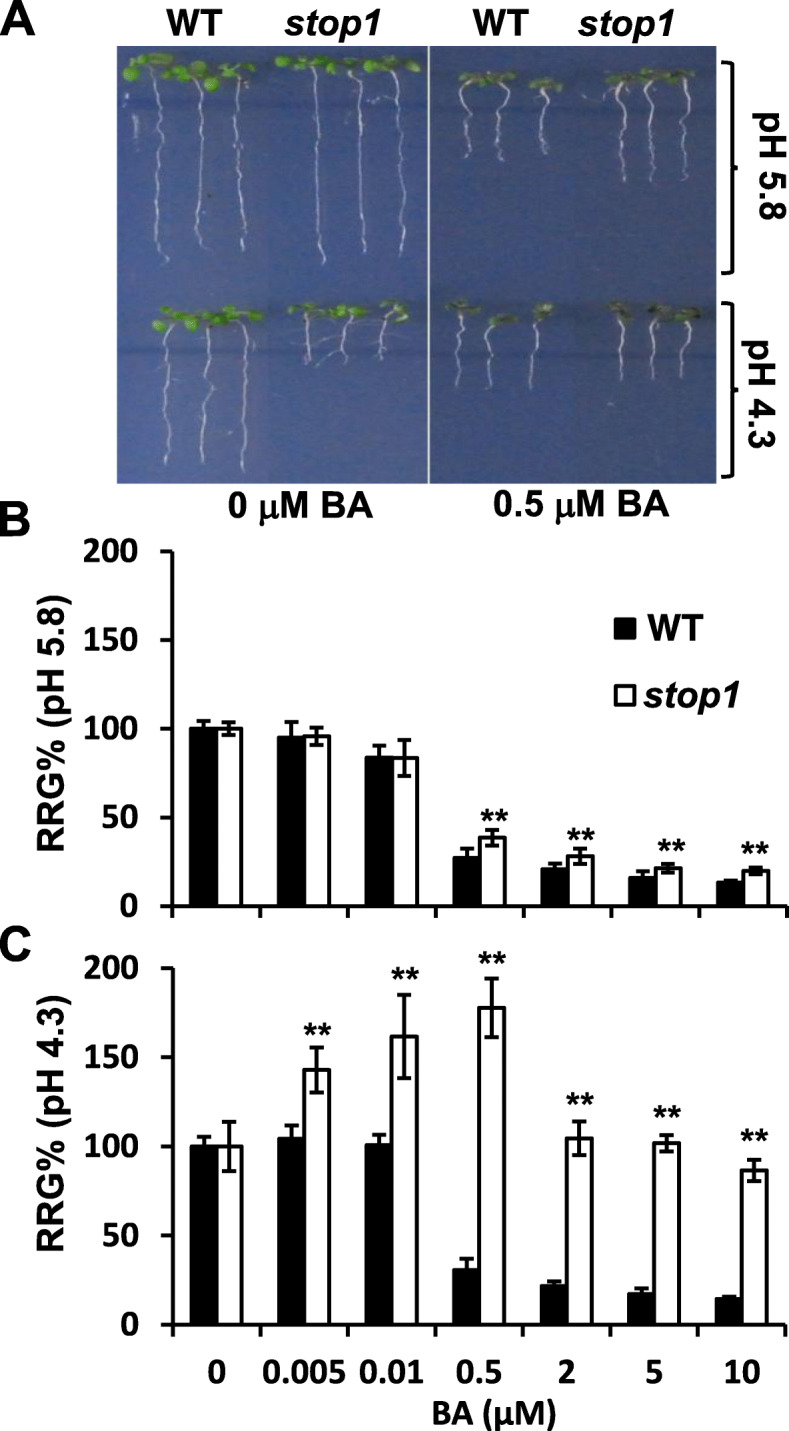
The *stop1* mutant is hyposensitive to cytokinins and is partially rescued by low concentrations of BA at pH 4.3. **A** Representative 5-d-old seedlings of WT and *stop1* germinated and grown on ½ x MS gellan gum plates (pH 4.3 or 5.8) supplemented with or without 0.5 μM BA. Relative root growth (RRG%) (+BA vs. –BA) of WT and *stop1* at pH 5.8 (**B)** and pH 4.3 (**C**). Data are means ± SD of three biological replicates. In each replicate, *n* = 20. Asterisks indicate significant differences (*p* < 0.01) between *stop1* and WT at indicated BA concentrations

At normal pH (5.8, + 0 μM BA), 5-d-old seedlings of the wild type (WT, *Col-0*) and the *stop1* null mutant (SALK_114180) exhibited a similar root-growth pattern (Fig. 1A, the left panel), confirming that *STOP1* is not required for normal plant growth (Iuchi et al., 2007). However, a low-pH treatment (pH 4.3, + 0 μM BA) reduced the WT root growth by 31% (Fig. 1A, the left panel) but inhibited root growth of the *stop1* mutant by ~ 95% (Fig. 1A, the left panel), confirming that the *stop1* mutant is highly susceptible to proton stresses (Iuchi et al., 2007; Sawaki et al., 2009). These results indicate that STOP1 plays an essential role in resistance to low-pH stresses, *i.e.*, proton stresses, in Arabidopsis.

Next, we examined the combinational effects of proton stresses and 6-benzyladenine (BA), an active cytokinin (Flores and Tobin, 1986), on root growth of WT and *stop1*. Under a pH 5.8 condition, root growth of the WT and the *stop1* mutant was inhibited comparably by low BA concentrations (≤0.01 μM) (Fig. 1B). The relative root growth (RRG%; +BA vs. –BA, at pH 5.8) of the WT and *stop1* plants was comparably decreased by ~ 5 and 16% in the presence of 0.005 and 0.010 μM BA, respectively (Fig. 1B). However, higher BA concentrations (≥0.5 μM, pH 5.8) caused more potent root-growth inhibition in the WT than in the *stop1* mutant (Fig. 1A, the right panel, and 1B). The root growth was decreased by 73 and 61% in the WT and *stop1* plants, respectively, in the presence of 0.5 μM BA at pH 5.8 (Fig. 1A, B). These results indicate that the *stop1* mutant was less sensitive to cytokinin-induced root-growth inhibition at pH 5.8.

Interestingly, the WT became insensitive to low concentrations of BA (≤0.01 μM), *i.e.*, RRG%s = 100%, at pH 4.3 (Fig. 1C), whereas the same low BA concentrations (≤0.01 μM) led to slightly reduced root growth of the WT plants at pH 5.8 (Fig. 1B). Furthermore, although higher BA concentrations (≥ 0.5 μM) also strongly inhibited WT root growth at pH 4.3 (Fig. 1C), the degrees of root-growth inhibition were less profound at pH 4.3 than at pH 5.8 (Fig. 1B, C). In WT, RRG%'s (+BA vs. –BA) decreased by 73% at pH 5.8 vs. 69% at pH 4.3 (Fig. 1B, C). These results indicate that proton stresses reduced the sensitivity of the WT to cytokinins.

More interestingly, at low pH (4.3), low BA concentrations (0.005, 0.01, and 0.5 μM) promoted root growth of the *stop1* mutant, leading to 1.4-, 1.6-, and 1.7-fold increases in RRG%'s (+BA vs. –BA, pH 4.3), respectively (Fig. 1A, C). However, when BA concentrations were ≥ 2 μM, the promotional effect of BA on root growth of *stop1* disappeared (Fig. 1C). These results further support the notion that proton stresses suppress the cytokinins’ inhibitory impacts on root growth in Arabidopsis.

### Involvement of proton stresses, STOP1 function, and cytokinins in controlling root-meristem size and root stem-cell identity

Root growth results from cell division in the root meristem and subsequent cell elongation in the root elongation zone. It has been demonstrated that cytokinins play crucial roles in regulating root-meristem activities (Dello Ioio et al., 2007). To investigate a possible interaction of low-pH stresses, STOP1 function, and exogenous cytokinins in controlling Arabidopsis root-meristem activities, we followed the root-meristem development of the WT and *stop1* mutant under different pH and cytokinin treatments.

**Fig. 2:**
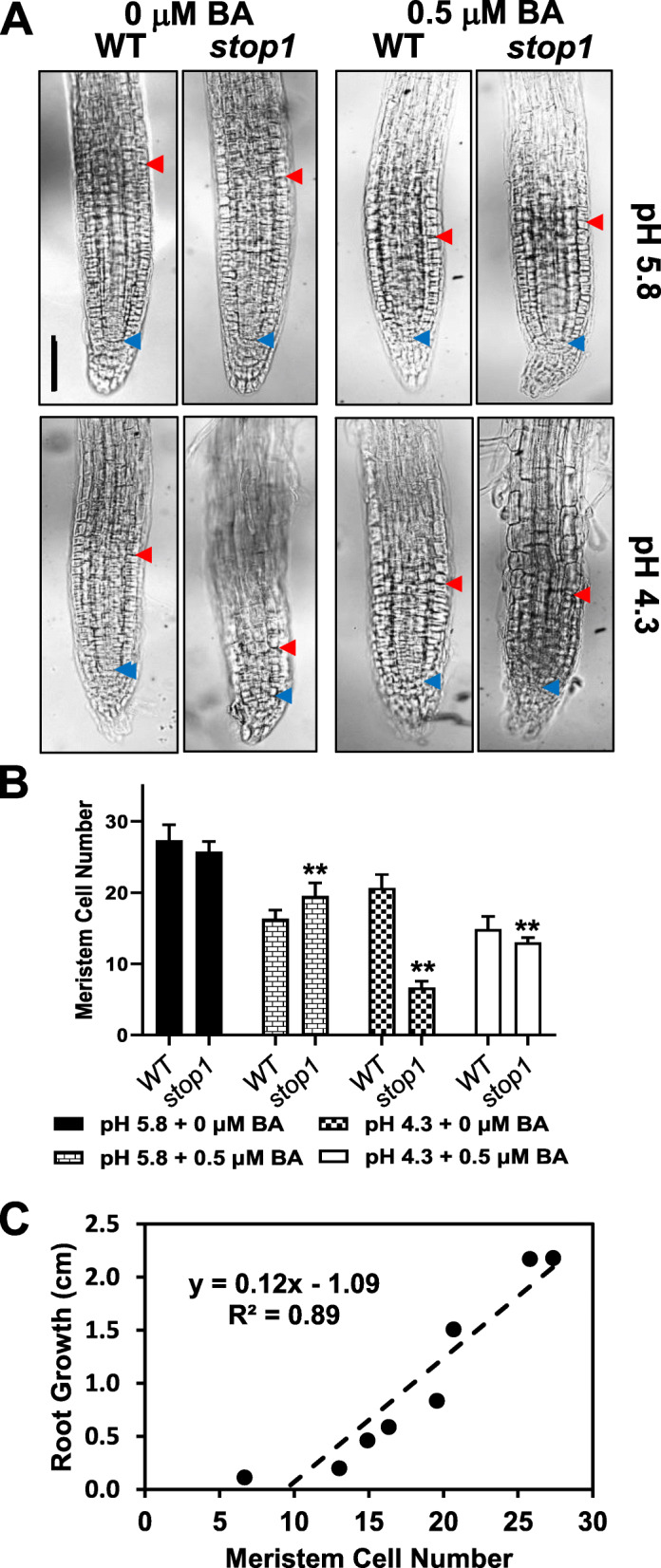
STOP1 function and low-pH stresses affect root meristem cell size (**A, B**). WT and *stop1* seeds were germinated and grown on ½ x MS agar plates for 4 d. Then, the 4-d-old seedlings were transferred to new ½ x MS gellan gum plates (pH 5.8 or 4.3) supplemented with or without 0.5 μM BA for a 16-h treatment. Root meristem size is defined as the number of cells from the quiescent center (QC) to the first elongated cortex cell. Data represent the mean of 25 plants (*n* = 25). **C** Linear regression analysis for root growth and meristem-stem cell number. Each dot represents the mean from ten plants of the WT or *stop1* under different treatments. Scale bar = 100 μm. The blue arrowhead point to the QC; the red arrowhead indicates the root transition zone (TZ) marked by the first elongated cortex cell. Asterisks represent significant differences between WT and *stop1* under indicated treatment concentrations (*P* < 0.01)

Root-meristem size, or root-meristem-cell number, is defined as the number of a file of cortex cells from the quiescent center (QC) to the first elongated cell (Fig. 2A) (Casamitjana-Martınez et al., 2003; Dello Ioio et al., 2007). The first elongated cell also marks the root transition zone (TZ) (Baluška et al., 2010). At pH 5.8 (−BA), the root-meristem size was comparable between the 4-d-old WT and *stop1* seedlings (Fig. 2A, B). However, a 16 h treatment of 0.5 μM BA significantly reduced root-meristem size in both lines at pH 5.8 (Fig. 2A, B). This result was consistent with the reported cytokinin’s action on root meristem size (Dello Ioio et al., 2007). In contrast, the exogenous cytokinin had significantly less influence on the root meristem size of the *stop1* mutant than the WT at pH 5.8 (Fig. 2A, B). The root meristem size decreased by 40% in WT versus 24% in *stop1* upon a 0.5 μM BA treatment at pH 5.8 (Fig. 2B). This result indicates that the loss-of-STOP1 function suppresses the inhibitory effects of cytokinins on root meristem activities.

At low pH (4.3) without an exogenous BA, the WT root meristem size decreased by 24% compared with that at pH 5.8 (−BA) (Fig. 2A, B). This result indicates that the low-pH stresses inhibit root meristem activities in Arabidopsis. However, the WT still maintained an intact root stem-cell niche at pH 4.3 (Fig. 2A; Fig. S2A). In contrast, the root meristem activity was severely suppressed in the *stop1* mutant at pH 4.3, decreased by 74% compared with the pH 5.8 (−BA) condition (Fig. 2A, B). Furthermore, the *stop1* mutant lost the root quiescent center (QC) and the root stem-cell niche, at pH 4.3 (−BA) (Fig. 2A; Fig. S2B). These results indicate that STOP1 is essential for maintaining root meristem activities and root stem-cell identity under low-pH stresses.

Interestingly, a 0.5 μM BA treatment improved root meristem activities of the *stop1* plant at pH 4.3 (Fig. 2A, B). The root meristem size of *stop1* increased 95% and the root stem-cell niche appeared with the BA treatment compared to the non-BA treatment condition at pH 4.3 (Fig. 2A, B; Fig. S2C). Therefore, the effect of 0.5 μM BA application on partially restored root growth of *stop1* at pH 4.3 (Fig. 1A, C) could be partly explained by the BA-mediated restoration of root stem cells and the improved root meristem activities.

Moreover, compared with the non-BA condition, a 0.5 μM BA treatment resulted in a 24% decrease in root meristem size of the WT plant at pH 4.3, compared with a 40% decrease at pH 5.8 (Fig. 2A, B). These results indicated that the low-pH stress reduced the inhibitory effect of exogenous BA on root meristem activities.

Next, we conducted a statistical analysis to investigate the relationships between root growth, root-meristem size, low-pH stresses, and STOP1 function. Regression analyses indicated that root growth was highly associated with the root-meristem size of the WT and *stop1* plants under different pH and BA treatments (*R*^2^ = 0.89), which means 89% of the root-growth variation could be explained by the variation of the root-meristem size (Fig. 2C). Therefore, our results suggest that root-meristem activities mainly determined the observed root growth under low-pH stresses.

### The* stop1* mutant produces more lateral roots and adventitious roots

We further evaluated the combinational effects of cytokinins, pH, and STOP1 functions on lateral-root and adventitious-root formation in Arabidopsis. At pH 5.8 (−BA), the lateral-root density (*i.e.*, lateral-root number per cm of the primary root of a plant) and adventitious-root density (*i.e.*, adventitious-root number per plant) of the 5-d-old WT seedlings were 0.1 and 0.4, respectively (Table 1). In contrast, the *stop1* plants possessed 0.8 and 1.2 lateral-root and adventitious-root density under the same condition, respectively, significantly higher than the WT (Table 1). These results indicated that the loss-of-function mutation of STOP1 promoted the lateral- and adventitious-root formation at pH 5.8.

**Table 1:** Lateral-root density (LRD) and adventitious-root density (ARD) of seedlings treated without (−) or with (+) 0.5 μM BA at pH 5.8 or 4.3 on agar plates at six days after germination (DAG)

	**Lateral root density (−BA)**			**Adventitious root density (−BA)**		
	**WT**	***stop1***	***P*** **value**	**WT**	***stop1***	***P*** **value**
**pH 5.8**	0.1 ± 0.2	0.8 ± 0.4	< 0.001	0.4 ± 0.5	1.2 ± 0.4	< 0.01
**pH 4.3**	0.7 ± 0.4	40.0 ± 4.7	< 0.001	0.5 ± 0.3	1.4 ± 0.5	< 0.01
	**Lateral root density (+BA)**			**Adventitious root density (+BA)**		
	**WT**	***stop1***	***P*** **value**	**WT**	***stop1***	***P*** **value**
**pH 5.8**	0.0 ± 0.0	0.0 ± 0.0	1	0.0 ± 0.0	0.8 ± 0.6	< 0.01
**pH 4.3**	0.0 ± 0.0	0.0 ± 0.0	1	0.0 ± 0.0	0.9 ± 0.3	< 0.001

Lateral root density is defined as the number of lateral roots per cm of a primary root of a plant. Adventitious root density is defined as the number of adventitious roots per plant. Data are means ± SD of three replicates. In each replicate, *n* = 10. The *t*-tests were conducted to compare the differences between WT and *stop1*. BA, 6-benzyladenine

Furthermore, compared with the pH 5.8 (−BA) treatment, the low-pH (4.3, −BA) treatment significantly increased the numbers of lateral-root density in both WT and *stop1* (Table 1). The lateral-root density increased to 0.7 and 40.0 in WT and *stop1*, respectively. These results indicated that compared with pH 5.8 (−BA), the low-pH (4.3, −BA) treatment caused a 7- and 50-fold increase in lateral-root density in the WT and the *stop1* mutant, respectively (Table 1). These results indicate that both the loss-of-function of STOP1 and low-pH stresses promote lateral root formation in Arabidopsis. However, the effect of low pH on lateral-root formation was much more profound in *stop1* than in WT.

For adventitious roots, a pH change from 5.8 to 4.3 slightly increased the adventitious-root density in both WT and *stop1* (Table 1). However, the *stop1* mutant possessed significantly higher adventitious-root density than the WT under both normal- and low-pH conditions. Taken together, these results indicated that the STOP1 function imposed a more profound impact on adventitious-root formation than the effect of pH changes.

### Suppression of lateral- and adventitious-root formation by cytokinins

We then proceeded to test the effects of cytokinins on the lateral-root and adventitious-root formation in WT and *stop1*. Supplementation of 0.5 μM BA to the growth medium abolished the lateral-root formation in WT under both pH 5.8 and 4.3 conditions (Table 1). In contrast, treatment of 0.5 μM BA slightly reduced the adventitious-root density to 0.8 and 0.9 in the *stop1* mutant at pH 5.8 and 4.3, respectively (Table 1). These results indicated that the loss-of-function of STOP1 partially offset the inhibitory effects of 0.5 μM BA on adventitious root formation.

### Low-pH stresses and STOP1 functions influence cytokinin signaling outputs of the Arabidopsis plants

The different responses of the WT and *stop1* mutant to exogenous BA at pH 5.8 and 4.3 (Figs. 1, 2; and Table 1) prompted us to investigate the possible involvement of cytokinin signaling in STOP1-mediated resistance to low-pH stresses. We used the cytokinin-sensitive two-component-output-sensor (new) (TCSn) reporter to monitor cytokinin-signaling outputs in Arabidopsis plants *in vivo*. The synthetic TCSn promoter contains B-type Arabidopsis response regulator (ARR)-binding motifs and a minimal 35S promoter, explicitly responding to cytokinins but not other plant hormones (Müller and Sheen, 2008; Zürcher et al., 2013).

**Fig. 3:**
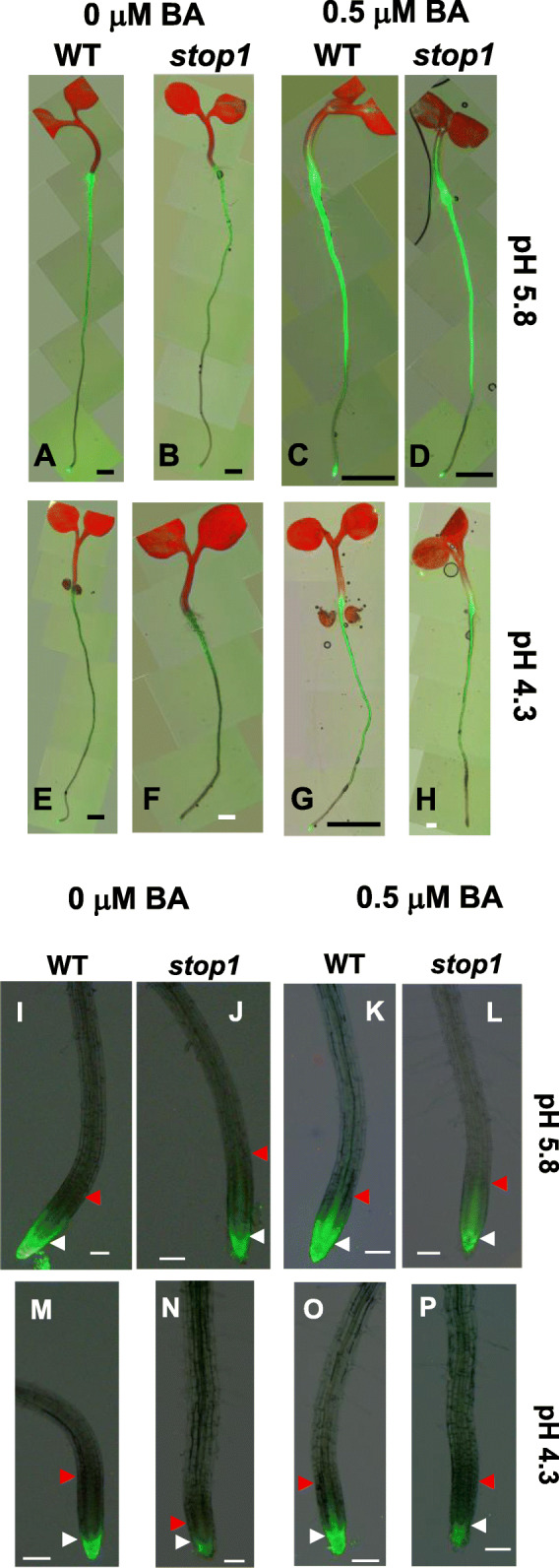
Cytokinin signaling outputs manifested by the TCSn:GFP fluorescence of 4-d-old seedlings (**A-H**) and their root tips (**I-P**) of the WT and *stop1*. Seeds were germinated on ½ x MS agar plates (pH 5.8) for 4 d. Then, 4-d-old seedlings were transferred onto ½ x MS gellan gum plates (pH 5.8 or 4.3) with or without supplementation of 0.5 μM BA for an additional 16-h treatment. Scale bars represent 0.1 cm for A-E, G; 0.01 cm for F, H; 100 μm for I-P

We examined the TCSn:GFP expression patterns in transgenic WT and *stop1* plants stably transformed with the TCSn:GFP construct. In general, 4-d-old WT and *stop1* seedlings shared the same tissue-specific TCSn:GFP expression patterns except that the TCSn:GFP expression intensity was weaker in the *stop1* mutant than in WT (Fig. 3).

At pH 5.8 (−BA), TCSn:GFP was expressed strongly in the root maturation zone with the most robust expression in the region near the root-shoot junction (Fig. 3A, B). Moreover, TCSn:GFP was strongly expressed in the root cap but weakly manifested in the stele precursor in the root meristem area (Fig. 3A, B, I, J).

At pH 5.8, a 16-h 0.5 μM BA treatment significantly enhanced the TCSn:GFP fluorescent intensity of the WT and *stop1* (Fig. 3C, D, K, L). Moreover, the TCSn:GFP expression was expanded to the root distal-elongation zone close to the root meristem region as well as the vasculature of the shoot (Fig. 3C, D). In addition, the TCSn:GFP expression was intensified in the root tip, especially in the root meristem region (Fig. 3C, D, K, L). However, the TCSn:GFP intensity in the root tip was weaker in *stop1* than in WT (Fig. 3C, D, K, L).

Interestingly, the low-pH (4.3, −BA) treatment significantly brought down the TCSn:GFP expression in WT and *stop1* (Fig. 3E, F, M, N) compared with that of the corresponding lines at pH 5.8 (−BA) (Fig. 3A, B, I, J). Furthermore, the TCSn:GFP fluorescence was weaker in *stop1* (Fig. 3B, F, J, N) than in WT (Fig. 3A, E, I, M) under the low-pH (4.3, −BA) condition.

At low pH (4.3), a 0.5 μM BA treatment led to enhanced TCSn:GFP fluorescence of the WT (Fig. 3G, O) and *stop1* (Fig. 3H, P) compared with the corresponding non-BA treatment (Fig. 3E, F, M, N). However, the TCSn:GFP fluorescence was weaker at pH 4.3 (Fig. 3G, H, O, P) than at pH 5.8 (Fig. 3C, D, K, L) and weaker in *stop1* (Fig. 3D, H, L, P) than in WT (Fig. 3C, G, K, O).

Taken together, the TCSn:GFP fluorescence intensity, or the cytokinin signaling output, is more robust in the WT than in the *stop1* mutant and stronger at pH 5.8 than at low pH. These results suggest that low-pH stresses and the loss-of-function STOP1 suppress cytokinin-signaling outputs in Arabidopsis.

### Expression of key genes involved in cytokinin biosynthesis, degradation, and signaling under low-pH stresses

To further address the roles of cytokinins in STOP1-mediated low-pH resistance, we examined the expression patterns of critical genes in the TCS system, including those encoding the enzymes for cytokinin biosynthesis (IPT3 and CYP735A2) and degradation (CKX1) as well as cytokinin receptors (AHK2, AHK3, and AHK4) and type-A Arabidopsis response regulators (ARRs).

Adenosine phosphate-isopentenyltransferases (IPT) and cytochrome P450 mono-oxygenases CYP735A1 and CYP735A2 are critical enzymes of cytokinin biosynthesis (Hirose et al., 2008). Cytokinin oxidase/dehydrogenases (CKX; EC 4.5.99.12) are primary enzymes responsible for irreversible degradation of cytokinins and the majority of metabolic cytokinin inactivity (Mok and Mok, 2001). The cytokinin receptors AHK2, AHK3, and AHK4 are partially redundant positive elements in cytokine sensing (Ferreira and Kieber, 2005; Brenner et al., 2012; Cheng and Kieber, 2014). AHK3 has been demonstrated to regulate the differentiation of root meristem cells in Arabidopsis (Perilli et al., 2013). In contrast, the type-A ARRs are the primary cytokinin-responsive elements that negatively influence the cytokinin signaling outputs (Hwang and Sheen, 2001; Kiba et al., 2003; To et al., 2004; Leibfried et al., 2005).

**Fig. 4:**
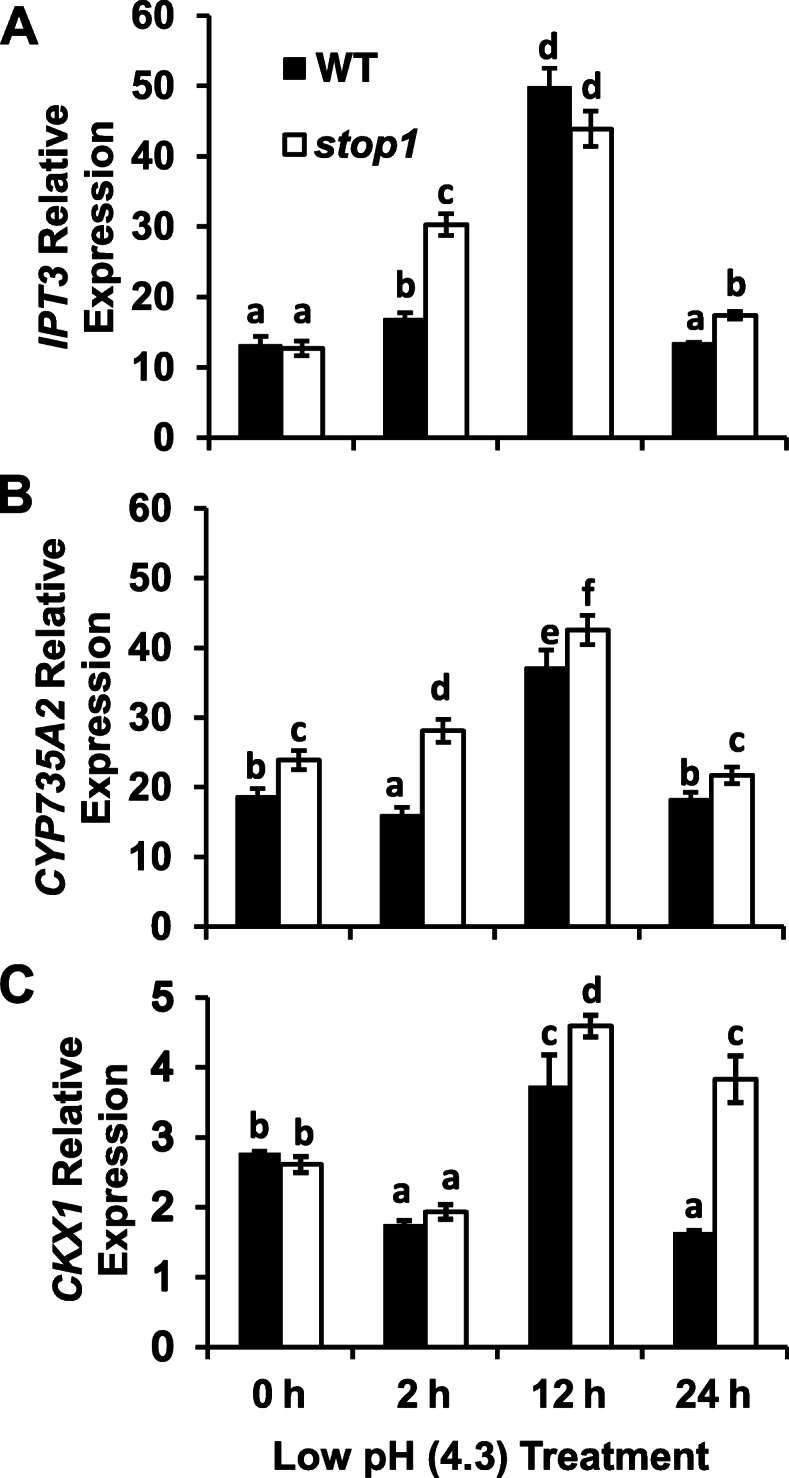
Relative expression of *IPT3* (**A**), *CYP735A2* (**B**), and *CKX1* (**C**) after low pH (4.3) treatment. Seeds were germinated and grown hydroponically in ½ x MS solution (pH 5.8) for 5 d before the seedlings were transferred to a fresh low-pH (4.3) hydroponic growth solution. Total RNAs were extracted from the root tissues for real-time RT-qPCR analyses. Data are means ± SD (*n* = 3). *, significant differences between the *stop1* mutant and WT (*p* < 0.05), **, highly significant differences between the *stop1* mutant and the WT (*p* < 0.01)

Real-time RT-qPCR analyses indicated that *IPT3* and *CKX1* expression levels in the root were comparable between WT and *stop1* under the control condition (0 h, pH 5.8) (Fig. 4A, C). However, *CYP735A2* expression was significantly higher in *stop1* than in WT under the control condition (0 h, pH 5.8) (Fig. 4B). The expression of *IPT3*, *CYP735A2*, and *CKX1* was significantly enhanced and peaked after 12 h low-pH (4.3) treatment in both WT and *stop1* (Fig. 4). However, the induction of the cytokinin-promoting genes, *IPT3* and *CYP735A2*, occurred much earlier, *i.e.*, at 2 h, in *stop1* than in WT whose strong *IPT3* and *CYP735A2* induction occurred at 12 h after the low-pH treatment (Fig. 4A, B). After peaked at 12 h after the low-pH treatment, *IPT3* and *CYP735A2* expression dropped back to their basal levels at 24 h in both WT and *stop1* (Fig. 4A, B).

In contrast, expression of the cytokinin biodegradation gene, *CKX1*, in the root was initially suppressed by 2 h low-pH treatment but strongly induced and peaked at 12 h in both WT and *stop1* (Fig. 4C). At 24 h, *CKX1* expression dropped back to its basal level in the WT but remained at a high level in *stop1* (Fig. 4C).

**Fig. 5:**
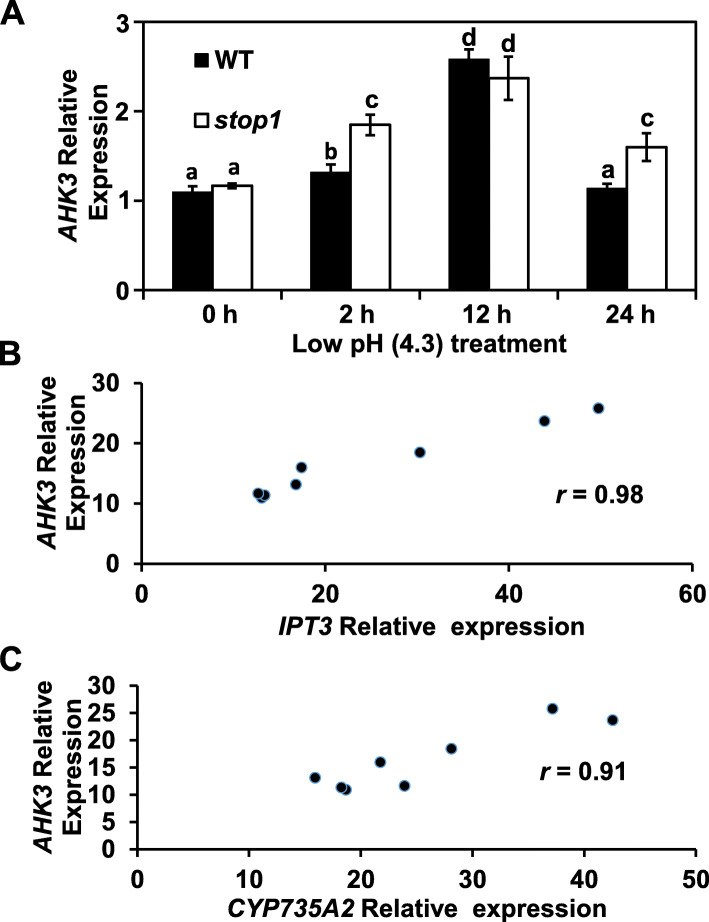
*AHK3* expression is induced by low pH (4.3, **A**) and is correlated with the expression of *IPT3* (**B**) and *CYP735A2* (**C**). Five-day-old seedlings germinated and grown hydroponically in ½ x MS solution (pH 5.8) were transferred to a new low-pH (4.3) hydroponic solution for indicated durations. Root tissues were collected for RNA extraction and subsequent real-time RT-qPCR analyses. Data are means ± SD (*n* = 3). *, significant differences between the *stop1* mutant and the WT (*p* < 0.05)

Under the control condition (pH 5.8, 0 h), the expression of the cytokinin receptor gene, *AHK3*, in the root was comparable between the WT and the *stop1* mutant (Fig. 5A). However, after 2 h low-pH treatment, *AHK3* expression in the root was quickly and strongly induced in *stop1* but was slightly enhanced in the WT (Fig. 5A). In addition, *AHK3* expression peaked at 12 h in both the WT and the *stop1* mutant (Fig. 5A). However, at 24 h, *AHK3* expression declined to its basal level in the WT but remained at a high level in *stop1* (Fig. 5A). These results indicated that under the low-pH (4.3) stresses, the *stop1* mutant had stronger and longer-lasting *AHK3* expression than the WT. Correlation analysis revealed that the expression levels of *AHK3* were highly and positively correlated with those of the other two cytokinin-promoting genes, *IPT3* and *CYP735A2*, with *r* = 0.98, 0.91, respectively (Fig. 5B, C). In contrast, the expression of the other two cytokinin receptor genes, *AHK2* and *AHK4*, was slightly induced by the low-pH treatment and showed no significant differences in gene expression levels between WT and *stop1* (Fig. S3).

**Fig. 6:**
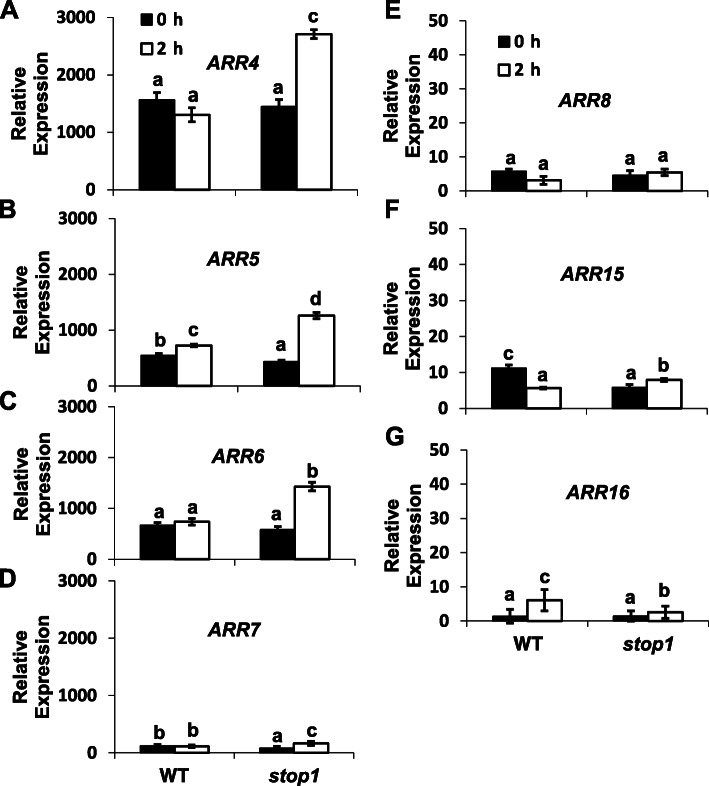
Relative expression of *ARR4* (**A**), *ARR5* (**B**), *ARR6* (**C**), *ARR7* (**D**), *ARR8* (**E**), *ARR15* (**F**), and *ARR16* (**G**) in WT and *stop1* with or without 2 h low pH (4.3) treatment. Five-day-old seedlings germinated and grown in ½ x MS solution (pH 5.8) were treated in a new low-pH solution for 2 h. Root tissues were collected for RNA extraction and subsequent real-time RT-qPCR analyses. Data are means ± SD (*n* = 3). Different letters indicate significant differences (*p* < 0.05) between different samples

To investigate the possible involvement of *ARR*s in cytokinin- and STOP1-mediated low-pH resistance, we examined the expression patterns of seven cytokinin-inducible type-A *ARR*s in the root. In general, under the control (pH 5.8, 0 h) condition, root expression of *ARR4*, *ARR5,* and *ARR6* was considerably higher than those of *ARR7*, *ARR8*, *ARR15,* and *ARR16* in the WT and the *stop1* mutant (Fig. 6).

Furthermore, in the WT, a low-pH (4.3) treatment significantly increased the expression of *ARR5* and *ARR16*, whereas the expression of the rest type-A *ARRs* either remained unchanged or slightly declined in the case of *ARR15* expression (Fig. 6). These results indicate that the low-pH treatment barely impacts the expression of the cytokinin up-regulated type-A *ARR*s in the WT.

In contrast, the low-pH treatment quickly induced the expression of *ARR4*, *ARR5,* and *ARR6*, the three highly expressed and cytokinin-inducible type-A *ARR*s, in the root of *stop1* (Fig. 6). Therefore, low pH mimics cytokinins in inducing root expression of *ARR4*, *ARR5,* and *ARR6* in *stop1*.

## Discussion

### STOP1 suppresses the unknown factors that negatively affect root growth and development under low-pH stresses

**Fig. 7:**
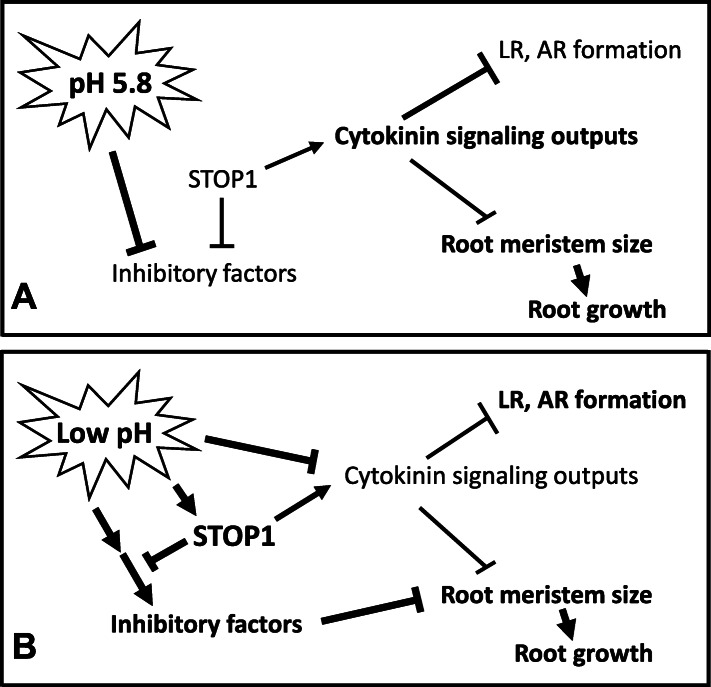
A working model for the STOP1-mediated and cytokinin-involved resistance to low-pH stresses. **A** The root-growth inhibitory factors are suppressed by normal pH. The STOP1 protein level remains low. The cytokinin signaling outputs are involved in defining the root meristem boundary, i.e.*i.e.*, the root meristem size, and inhibiting lateral root and adventitious root formation. The root meristem size is a key factor influencing root growth. **B** The root growth inhibitory factors are activated at low pH, which strongly suppresses root meristem size. STOP1 is activated by proton stresses, presumably at posttranslational levels, which represses the inhibitory factors but promotes the cytokinin signaling outputs. Arrows indicate promotion. T bars indicate inhibition

It has been well documented that STOP1 is regulated at posttranslational but transcriptional levels in Arabidopsis. For instance, *STOP1* transcript levels remained unchanged upon low-pH stresses (Iuchi et al., 2007). However, the STOP1 protein level remains low under normal growth conditions (pH 5.8) but is significantly enhanced under the low-pH and aluminum stresses via posttranslational regulations (Zhang et al., 2019; Fang et al., 2020). In this report, we demonstrated that STOP1 promoted cytokinin signaling outputs, influencing root meristem size and lateral and adventitious root formation (Figs. 2, 3; Table 1). Therefore, the lower STOP1 level at the normal pH will presumably contribute less to promoting cytokinin signaling outputs in the root as proposed in a working model for the STOP1-mediated and cytokinin-involved low-pH resistance network in Arabidopsis (Fig. 7A). In the root, cytokinins act antagonistically with auxins to define the boundary of auxin maximum, *i.e.*, the border of the root meristem in the root tip, and delimit the positions of lateral roots along the primary root and the adventitious roots on the stem (Werner et al., 2003; Ioio et al., 2008; Bielach et al., 2012). Thus, at normal pH, root cell division and differentiation activities are well balanced by the interactions between auxins and cytokinins.

The *stop1* null mutant is highly susceptible to proton stresses (Fig. 1A) (Iuchi et al., 2007; Jiang et al., 2017), indicating that STOP1 is essential for suppressing the root-growth inhibitory factors, which are turned on upon the proton stresses (Fig. 7B). In the meantime, low-pH (4.3) stresses also inhibited root growth of the WT plant (Fig. 1A), indicating that the STOP1-mediated low-pH resistant mechanism could not wholly offset the deleterious effects of the proton stresses (Fig. 7B).

Low-pH stresses inhibit root growth mainly via reducing the root meristem size, *i.e.*, the root meristem activity, in WT and *stop1* (Fig. 2). The reduced root meristem size could result from the unknown low-pH inhibitory factors and enhanced cytokinin signaling outputs, both of which are regulated by STOP1 (Figs. 1; 2A, C; 7B). In addition, STOP1 suppresses the unknown inhibitory factors activated by low-pH stresses (Fig. 7B), as indicated by the highly sensitive phenotype of the *stop1* mutant to proton stresses (Fig. 1).

The enhanced formation of lateral root and adventitious roots in *stop1* (Table 1) also suggests that the cytokinin signaling outputs were lowered in the *stop1* mutant. In other words, the loss of the wild-type function of STOP1 leads to suppressed cytokinin signaling outputs in the root (Fig. 7). Thus, the cytokinin signaling outputs were suppressed by low-pH stresses but promoted by STOP1 (Figs. 2, 3, 7B).

The overall cytokinin signaling outputs, reflected by the TCSn:GFP intensity, were lower at low pH than at normal pH (Figs. 2, 3, 7). These results indicated that, at low pH, the effect of low-pH stresses on suppressing cytokinin signaling outputs outperformed that of the STOP1 on promoting the cytokinin signaling outputs (Figs. 3A, E, I, M; 7B). As a result, the overall cytokinin signaling outputs in the roots were decreased at low pH. Furthermore, under the low-pH stresses, the root meristem activities could be promoted by the reduced overall cytokinin signaling outputs but suppressed by the unknown low-pH inhibitory factors (Figs. 1, 2, 7B). The potent root-growth-inhibition phenotypes of the WT and *stop1* plants under low-pH stresses indicate that the effects of the inhibitory factors exceed those of the reduced cytokinin signaling outputs in determining root meristem activities (Fig. 7B).

Furthermore, at low pH (pH 4.3), the *stop1* root lost its quiescent center (QC) identity (Figs. 2A, S2B). These results indicate that STOP1 is not only required for maintaining root meristem activities, but it also is essential for preserving the root stem-cell niche under proton stresses. However, further studies are necessary to elucidate the molecular bases underlying STOP1’s functions in these processes.

### Cytokinins are involved in STOP1-mediated resistance to proton stresses

Cytokinin oxidase/dehydrogenase 1 (CKX1) facilitates the breakdown of cytokinins in the plant cell (Werner et al., 2003). *CKX1* overexpression in Arabidopsis lowered endogenous cytokinin concentrations of the transgenic plants to 30–45% of the wild-type contents, suppressing shoot growth and development but promoting root growth and the formation of lateral and adventitious roots (Werner et al., 2003). These results indicate that cytokinins are positive and negative regulators for the shoot and root development, respectively (Werner et al., 2003; Aloni et al., 2006; Dello Ioio et al., 2007; Chang et al., 2013).

The WT seedlings produced significantly more lateral and adventitious roots at low pH than at normal pH (Table 1). Moreover, under the control condition (pH 5.8, −BA), the *stop1* seedlings possessed significantly more lateral and adventitious roots than the WT (Table 1). These phenotypes mimic the effect of reduced cytokinin-signaling outputs on promoting lateral and adventitious root formation (Nishimura et al., 2004; Bielach et al., 2012). Significantly, adding 0.5 μM BA to the growth media diminished or eliminated the promotional effects of the low-pH stresses and loss-of-STOP1 functions on promoting lateral-root and adventitious-root formation (Table 1). Therefore, the enhanced lateral- and adventitious-root formation by low-pH stresses and *stop1* could result from reduced cytokinin signaling outputs.

Indeed, our results indicate that the wild-type function of STOP1 boosts cytokinin signaling outputs in the root (Fig. 3), which could lead to suppressed lateral and adventitious root formation (Table 1). In the meantime, low-pH stresses suppress root cytokinin-signaling outputs (Fig. 3), positively influencing lateral and adventitious root formation (Table 1). Thus, at low pHs, the STOP1 function counteracts the inhibitory effects of low-pH stresses on reducing the cytokinin signaling outputs in the root (Fig. 7), which is essential for preserving root stem-cell identity and root meristem activities upon proton stresses for a reason explained below.

### Expression patterns of genes involved in the two-component signaling pathway

The expression of the cytokinin-promoting genes *IPT3*, *CYP35A2*, and *AHK3* was induced by low-pH stresses in both the WT and the *stop1* mutant (Figs. 4, 5), which could be a compensatory response to the lowered cytokinin-signaling outputs at low pH (Fig. 3). Furthermore, the expression of these cytokinin-promoting genes was induced by the low-pH stresses earlier in *stop1* than in WT (Figs. 4, 5). In contrast, the cytokinin-biodegradation gene *CKX1* was induced later than the cytokinin-promoting genes, and the steady-state of *CKX1* expression lasted longer in *stop1* than in WT (Fig. 4). These results are consistent with lower cytokinin signaling outputs in *stop1* than in WT under proton stresses (Figs. 2, 3), such that the *stop1* mutant requires a higher cytokinin compensation than the WT.

Type-A ARRs are negative regulators of the cytokinin signaling network, responsible for the final realization of the cytokinin signaling (Hwang and Sheen, 2001; Kiba et al., 2003; To et al., 2004; Leibfried et al., 2005). We found that the expression of three cytokinin-inducible type-A *ARR*s, *i.e.*, *ARR4*, *ARR5,* and *ARR6*, was highly induced by low pH stresses in *stop1* but not in WT (Fig. 6), which could contribute to the reduced cytokinin signaling outputs observed in *stop1* under proton stresses (Figs. 2, 3).

### Basal levels of cytokinins are required for root growth and development

It has been reported that the cytokinin signaling mutants, *ahk3* and *arr1, 12*, with reduced sensitivity to cytokinins (Dello Ioio et al., 2007) and the cytokinin deficient mutants with ~ 30–45% of the wild-type levels of cytokinins (Werner et al., 2003) exhibit accelerated root growth and enhanced formation of lateral and adventitious roots. These results indicate that cytokinins are negative regulators for root growth and development. However, higher-order loss-of-function mutants of the genes encoding cytokinin receptors (AHKs) and Arabidopsis His Phosphotransfer Proteins (AHPs) display reduced root meristem activities (Higuchi et al., 2004; Nishimura et al., 2004; Hutchison et al., 2006), indicating that although high cytokinin concentrations inhibit root growth, basal levels of cytokinin signaling outputs are essential for root meristem function (To and Kieber, 2008).

As discussed above, low-pH stresses and the loss-of-STOP1 function additively suppress the cytokinin signaling outputs in *stop1* (Fig. 7). As a result, the cytokinin signaling outputs in the *stop1* mutant could be too low to maintain the root stem-cell niche and root meristem activities under low pH stresses (Fig. 2A, B). This hypothesis is supported by the root phenotype of the WT at low pH (4.3, −BA) and the root phenotype of *stop1* at low pH with 0.5 μM BA. Figures 2A and S2B show that the root stem-cell niche was lost in the *stop1* mutant, whereas the WT maintained its QC and root stem-cell identity under proton stresses (Fig. S2). Furthermore, supplementation of 0.5 μM BA could rescue the root stem-cell identity, as evidenced by the restored QC structure in the root tip (Fig. S2C), and promoted root meristem activities of the *stop1* plant at low pH (Fig. 2A, B). These results suggest that the severe defects in root development and growth of the *stop1* plant are partially due to its extremely low cytokinin signaling outputs in the root. Therefore, it is necessary for STOP1 to counteract the effects of low-pH stresses on reducing cytokinin signaling outputs under proton stresses (Fig. 7B).

## Materials and methods

### Plant materials and growth conditions

The wild-type *Arabidopsis thaliana* (Columbia ecotype, Col-0) and the *stop1* T-DNA insertion line, SALK_114180 (*stop1*) were acquired from the Arabidopsis Biological Resource Center (ABRC). Seeds were surface sterilized with 70% (v/v) ethanol for 10 min and washed 5 times with sterilized water. After 2 d of cold treatment (4 °C), seeds were sown on half-strength (1/2 x) Murashige and Skoog (MS) medium plates (1.2% agar, 1% sucrose, pH 5.8) and grown in a growth chamber with a 16 h: 8 h, light: dark, cycle at 23 °C (Hoekenga et al., 2006; Jiang et al., 2017; Wang et al., 2017; Wang et al., 2018; Wang et al., 2021). After 4 d, seedlings were transferred to 1/2 x MS plates (0.8% gellan gum, 1% sucrose, pH 5.8 or 4.3) with different concentrations of 6-benzyladenine (BA) (Sigma-Aldrich) and grown in the same growth chamber. Then, root growth of 5-d-old seedlings was measured. For hydroponic growth, seeds were germinated and growth on 1/2 x MS solutions at pH 5.8 or 4.3 for 5 d.

### Measurement of lateral root density and adventitious root density

Total lateral roots and adventitious roots were counted for individual 6-d-old seedlings germinated and grown on 1/2 x MS medium (pH 5.8 or 4.3) plates supplemented without or with 0.5 μM BA. Lateral root density was defined as the number of lateral roots per cm of the primary root, and adventitious root density was defined as the number of adventitious roots per plant.

### Root length and meristem size analyses

Seeds were germinated and grown on 1/2 x MS agar plates (pH 5.8) in a growth chamber with 16 h: 8 h, light: dark (23 °C) for 4 d. Then, the 4-d-old seedlings were transferred to 1/2 x MS gellan gum plates (pH 5.8 or 4.3) supplemented without or with 0.5 μM BA for a 16-h treatment in a growth chamber with 16 h: 8 h, light: dark (23 °C). The treated seedlings were then cleared as described (Malamy and Benfey, 1997). Subsequently, the cleared root samples were observed with a DM5500B Leica Differential Interference Contrast (DIC) microscope under a 20x water immersion objective. Each experiment contained a minimum of 25 plants. Experiments were repeated twice independently. Root meristem size is defined as the number of cortex cells in a file from the quiescent center to the first elongated cortex cell (Perilli and Sabatini, 2010). For measuring root length, plates were photographed. Then, the root images were analyzed by the Image J software (https://imagej.nih.gov/ij/).

### Monitoring cytokinin-signaling outputs with the TCSn:GFP reporter

The *Two-Component signaling Sensor* new (TCSn):*green fluorescent protein* (GFP) construct (Zürcher et al., 2013) was stably transformed into the WT (Col-0) and the *stop1* mutant. Transgenic TCSn:GFP lines (T2) exhibited strong GFP expression patterns that match the known cytokinin responses and functions were chosen for further studies. Four-day-old seedlings germinated on vertical half-strength (1/2 x) MS agar plates (pH 5.8) were transferred to 1/2 x MS gellan gum plates (pH 4.3 or 5.8), supplemented with or without 0.5 μM BA for a 16 h treatment (Jiang et al., 2017). Subsequently, the seedlings and their GFP fluorescent patterns were observed under a Leica Laser Microdissection 7 (LMD7) microscope.

### RNA isolation and real-time quantitative RT-qPCR

About 10 mg of surface-sterilized seeds were germinated in Magenta boxes containing sterile hydroponic growth solution (1/2 x MS) (pH 5.8) in a growth chamber with a 16 h light: 8 h dark cycle at 23 °C (Hoekenga et al., 2006; Wang et al., 2017). After 5 d, seedlings were treated with new low-pH (4.3) hydroponic growth solutions for indicated durations. No differences in responses of the WT and *stop1* plants to low-pH stresses were found when the treatments were conducted on agar plates or in hydroponic solutions (Fig. S1).

Total RNAs were extracted from root tissues with the RNeasy Mini Kit (Qiagen) according to the manufacturer’s instructions. First-strand cDNAs were synthesized from 5 μg DNase I-digested total RNAs using the SuperScriptIII First-Strand Synthesis System (Invitrogen). Real-time RT-qPCR was performed with a 7500 Fast Real-Time PCR System (Applied Biosystems, Inc.) according to manufacturers’ protocols. The relative expression levels of the target genes were referred to an endogenous calibrator gene, 18S rRNA. The sequences of the RT-qPCR primers are listed in Table S1.

## Supplementary Information

**Additional file 1: Fig. S1.** The effects of agar and hydroponic media on the root growth of wild type and *stop1*. **Figure S2.** The loss of root stem-cell niche in *stop1* under low-pH stresses. **Figure S3.** The effects of low pH treatment on expression of *AHK2* and *AHK4*. **Table S1.** Sequences of real-time RT-qPCR primers.

## Data Availability

The data supporting this study’s findings are available from the corresponding author, JL, upon reasonable request.

## Abbreviations

AHK: Arabidopsis Histidine Kinase
AHP: Arabidopsis His phosphotransfer Protein
ALMT1: Aluminum-activated malate transporter 1
ALS3: Aluminum sensitive 3
ARR: Arabidopsis response regulator
BA: 6-benzyladenine
CKX: Cytokinin oxidase/dehydrogenases
CYP735A2: Cytochrome P450 monooxygenase 735A2
GUS: β-glucuronidase
IPT3: Isopentenyl transferase 3
MATE: Multidrug and toxic compound extrusion
RR: Response regulators
RRG%: Relative root growth
STOP1: Sensitive to proton rhizotoxicity1
TCS: Two-component signaling
