# Supplementary material for: Involvement of cytokinins in STOP1-mediated resistance to proton toxicity
Source: Stress Biol. 2022 Mar 8;2(1):17. doi: 10.1007/s44154-022-00033-6 (PMC10441851; doi:10.1007/s44154-022-00033-6)
Supplement: Supplementary file 1 — Additional file 1: Fig. S1. The effects of agar and hydroponic media on the root growth of wild type and stop1. Figure S2. The loss of root stem-cell niche in stop1 under low-pH stresses. Figure S3. The effects of low pH treatment on expression of AHK2 and AHK4. Table S1. Sequences of real-time RT-qPCR primers. [file 44154_2022_33_MOESM1_ESM.docx]

**Supplementary materials**


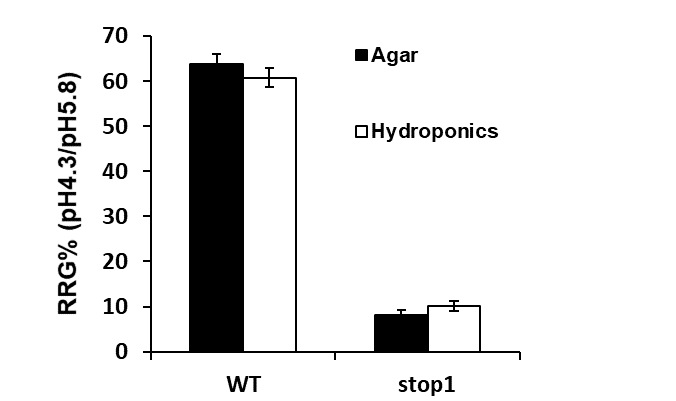


***WT stop1***

**Fig. S1.** Comparable results from the agar plate and hydroponic treatments. Seeds of the wild type (WT) and the *stop1* mutant were germinated and grown on agar plates or in hydroponic growth solutions (1/2 x MS) at pH 4.3 or 5.8 for 5 d. Relative root growth (RRG%) was calculated as the growth of primary roots at pH 4.3 over at pH 5.8. Data are means ± SD of three biological replicates. In each replicate, n =10.


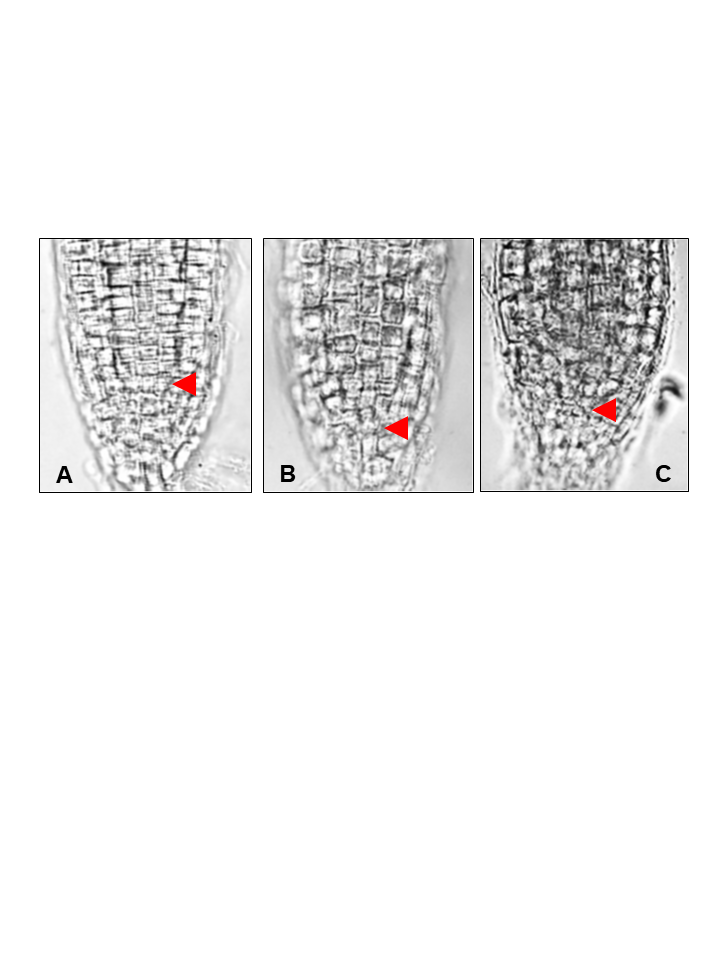


**Fig. S2.** The root tips of the 4-d-old *stop1* seedlings under the 16-h treatment of normal pH (5.8) (A), low pH (4.3) (B), or low pH (4.3) with supplementation of 0.5 μM BA (C). The arrowhead points to the root quiescent center (QC).


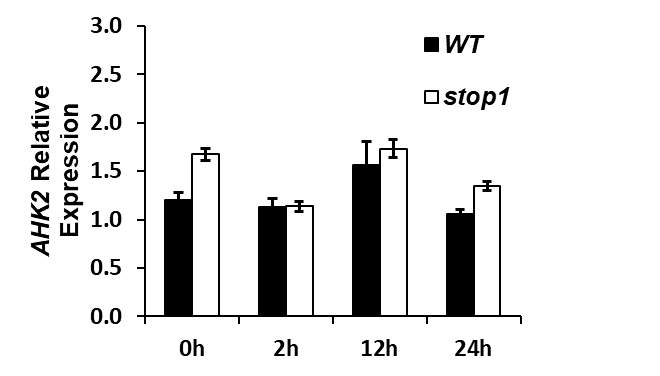


**A**


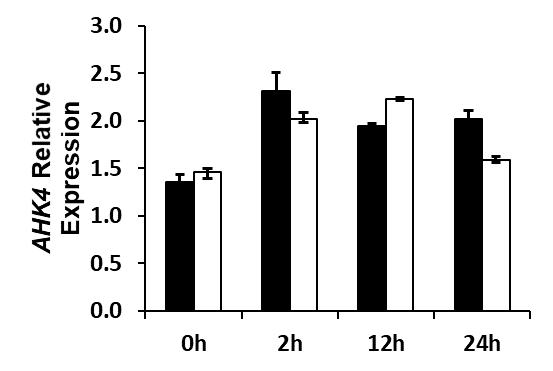


**B**

**Low pH (4.3) treatment**

**Fig. S3.** Relative expression of *AHK2* (A) and *AHK3* (B) in the root after low pH (4.3) treatment. Five-day-old seedlings germinated and grown hydroponically in ½ x MS solution (pH 5.8) were transferred to a new low-pH (4.3) hydroponic solution for indicated durations. Root tissues were collected for RNA extraction and subsequent real-time RT-qPCR analyses. Data are means ± SD (n= 3).

**Low pH (4.3) treatment**

**Table S1. Sequences of real-time RT-qPCR primers.**

| **Gene Name** | **Forward Primer** | **Reverse Primer** |
| --- | --- | --- |
| ***AHK4*** | TAGCGATGACTGCGGATGTGATAC | AAACTCGCCGGAAGATTGGATTA |
| ***ARR4*** | TCGACGGCAGAAGATGAG | ACGAAGTTAACGGAGATAGA |
| ***ARR5*** | ATGTCCTGATTCTTTCGGCTTAC | AACCCATCTTTGTCACTCTTG |
| ***ARR6*** | TACAATTTTCCAACCCCTATG | AAAAAGCTATGACCCCTCTAA |
| ***ARR7*** | TATGTCACCTAACCTAACTTC | ACCGCCATTGTCAAACTCAGA |
| ***ARR8*** | TAGGGAAGGTAGCGAGTATTGTA | AACGTCCCATCCCCTTATTGTC |
| ***ARR15*** | CCTCCCTTGATGACGACACT | TTACACAAGGAAATCAAGAGA |
| ***ARR16*** | ATCTCTTCCCCATTGCTCTC | TCTCTGGTGATGGTATTTATGATG |
| ***IPT3*** | GTCTAGTGGCGTGATCAGTTGTAA | ATCATTGCCCCGTCACCATCAC |
| ***CYP735A2*** | ACAATGTCCGCGATGAGGTC | GTAAAAGTGTGGCAGGAGGGTAAA |
| ***AHK2*** | GAGAGCTTTTTGACATCGGGTAG | TCGGTTTTCGCAGTTTTTCTCAC |
| ***AHK3*** | GAAGTGTGGAATGGATGGGTATG | GTTTTTGTGTTTCCTTCGCTGTTC |
| ***CKX1*** | TCAATTGGGTCCACGAATCTCGTT | AGGCGCCAAATTTCTGCCATCT |
| ***18S rRNA*** | CGCTATTGGAGCTGGAATTACC | AATCCCTTAACGAGGATCCATTG |
